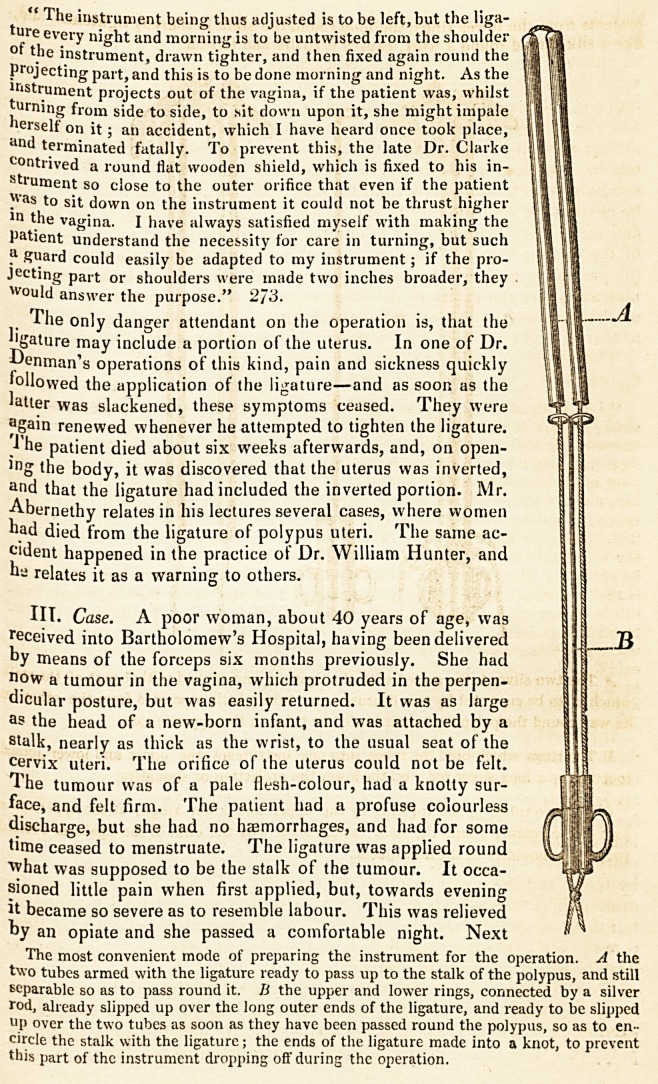# Dr. Gooch on Polypus of the Uterus

**Published:** 1830-04-01

**Authors:** 


### ON POLYPUS OF THE UTERUS. By *Robert Gooch*, M.D.

[Diseases of Women, Chap. V.]

THE above is the only subject which we have left unnoticed in Dr. Gooch's valuable volume, and we now proceed to close our analytical labours, as far as the talented author is concerned—at least till he again makes his appearance on the literary stage, which, we hope may be soon.

The experienced author believes that polypus of the uterus is a more frequent disease than is commonly supposed—and that those who, in extensive practice, have not met with it, must have overlooked it. If mistaken and neglected, it occasions the death of the patient—if detected and removed, she lives and regains perfect health. The cure of polypus uteri affords one of the most striking instances of the triumph of art over the infirmities of nature.

"This disease is commonly for a long time mistaken for profuse menstruation, the patient instead of menstruating regularly and moderately, has frequent and profuse hæmorrhages from the uterus, and in the intervals a pale discharge. These gradually drain her circulation and injure her health, until she acquires the deadly paleness and suffers the complaints which are the ordinary effects of deficiency of blood. The absence of pain in the uterus or pelvis, (for there is often none, and never that degree which attends the malignant diseases of this organ,) leads to no suspicion that the hæmorrhages depend on a disease of structure. Tonics and astringents are given in various forms; one practitioner is consulted after another, till at length the uterus is examined, and a polypus is discovered. This is the history of most of the cases which I have met with.

"A polypus of the uterus, when discovered, is a tumour in the vagina attached to some part of the uterus. It is round, smooth, firm, and insensible; it is quite unattached to the vagina, so that the finger can be passed round between the walls of the vagina and the surface of the tumour; but if traced higher up, it is found to terminate in a narrower part or stalk. This stalk is differently attached in different cases; in some it passes through the orifice of the uterus into its cavity, and is attached to the fundus of this organ; in others, it passes into the cavity of the neck, to one side of which it is attached; in others it does not enter the orifice, but is attached to one portion of its edge or lip; hence a distinction of polypus of the fundus, polypus of the neck, and polypus of the orifice. This distinction must not be lost sight of, for it is of practical consequence. In ascertaining the nature of the tumour for the purpose of determining the propriety of removing it by an operation, the mode of its attachment is one of our chief guides; and in this respect what is true of polypus of the fundus, is not so of polypus of the neck or lip.

"In polypus of the fundus the stalk is completely encircled by the neck of the uterus, and if the finger can be introduced into the orifice, it passes easily round between the stalk of the polypus and the encircling neck.

"In polypus of the neck the finger cannot be passed quite round the stalk; it may be passed partly round it, but it is stopped when it comes to that part where it is attached to the neck, the stalk is only *semi*-circled by the neck.

*A* Polypus of the *fundus*, its stalk growing from that part of the uterus, its body down in the vagina, the lower part of its stalk surrounded by the orifice of the uterus.

*C* Polypus of the *neck* of the uterus, its stalk growing from the cavity of the neck, and consequently only semi-circled by the orifice, its body down in the vagina.

"In polypus of the edge of the orifice or lip, the stalk does not enter the orifice, but grows from the edge of it; it feels as if a portion of the lip was first prolonged into the stalk, and then enlarged into the body of the polypus. It is important to remember that there is a polypus, the stalk of which is not encircled by the orifice of the uterus; if it grows from the orifice it cannot be encircled by it." 254.

When a polypus grows within the uterus, it dilates its cavity, neck, and orifice, as in pregnancy. The orifice is a round space, with thin edges, as in somewhat advanced pregnancy, instead of having a projecting part of the neck forming a narrow chink in a firm thick nipple. In polypus of the neck and that of the lip, the projecting part of the uterus preserves more of its ordinary form and consistence.

"The internal structure of polypus, in most cases, exactly resembles the internal struture of the *large white tubercle* of the uterus, commonly called the fleshy tubercle; 'so that a person looking on a section of the one and the other, out of the body, could not distinguish between them.' They are the same disease, differing only in the seat and mode of their attachment, and consequently in the symptoms which they produce. On cutting into them we see a hard whitish substance intersected by membranous partitions. This, however, is not always its structure, it is sometimes of a much softer and looser consistence, and sometimes has considerable cavities." 255.

*B* Polypus of the *orifice* or *lip* of the uterus; the orifice on the front of the stalk, and not at all encircling it, the body of the polypus down in the vagina.

The external covering of the polypus is the internal covering or mucous membrane of the uterus. When the patient is cured by the removal of the polypus, it comes away in a putrid state, unfit for a minute anatomical examination; but sometimes the patient dies before the nature of the case is discovered, and then the tumour can be examined attached to the body and unchanged by an operation.

The size of polypi differs very much in different cases. Dr. G. has removed several as large as the head of a new-born child; but they are commonly of a much less size, and he has known several cases where frequent hæmorrhages were occasioned by a polypus not larger than a filbert, attached just within the cavity of the cervix. The hæmorrhage ceased on the removal of the polypus.

Thus then a polypus of the uterus is commonly a round insensible tumour, growing by a stalk from the fundus, cervix, or lip, in its inner structure like a fleshy tubercle—on its outside covered by a mucous membrane of a pale flesh colour, streaked with veins, and occasioning frequent hæmorrhage from the uterus. When the polypus grows from the fundus of the uterus, it is at first, very small, resides within the cavity of this organ, and, for some time occasions no uneasiness or disturbance in its functions, that might lead to suspicion of its existence. But as the polypus grows larger it gradually dilates the uterus, till at length this organ, stimulated by its bulk, begins to contract upon it, protruding it through the dilated orifice. It sometimes passes through the orifice gradually and insensibly—sometimes suddenly during the action of the bowels. Dr. G. has known several instances where retention of urine has followed this sudden descent of the polypus, from compression of the urethra. While the tumour remains within the uterus, it cannot be felt in a common examination, and the nature of the disease is generally overlooked. The following is a striking instance, which we shall give in the author's own words.

"A lady who had been subject to frequent and profuse hæmorrhages from the uterus, had consulted two eminent practitioners in Edinburgh, without relief. The uterus was examined, but no change of structure was discovered. As she passed through London on her way to the continent, she consulted me. I examined the uterus, and discovered nothing. She went to Rome, and then to Geneva, at both which places she consulted some of the most eminent practitioners, by whom the uterus was examined, but nothing was discovered, excepting that it was rather larger than natural. Thus far the disease was considered as common menorrhagia. After being absent from England a year and a half, she returned to London worse than when she went. A few days after her arrival, she had a recurrence of the hæmorrhage, but it was attended by an unusual symptom; the blood came away in large coagula attended with bearing down pains. As soon as the hæmorrhage had ceased, I advised her to allow me to examine the uterus, though I little expected what I was to find. She consented, and the next morning I visited her at her hotel before she was up. As I passed my hand under the bed-clothes, I said I fully expect to find nothing, yet the next instant I had my finger on a polypus; it was about the size of a large walnut, with a slender neck encircled by the orifice of the uterus. It was easily removed, the hæmorrhages have never returned, and she has since enjoyed perfect health. There can be no doubt that the polypus was expelled into the vagina during the pains which attended the last hæmorrhage, and that I had the rare good luck to examine almost immediately after the tumour was discoverable. If I had examined before those pains, I should have overlooked the disease, as I, as well as her attendants at Edinburgh, Rome, and Geneva, had done before." 260.

When polypus of the fundus descends into the vagina, the stalk drags downwards that portion of the fundus to which it is attached, so that, in this stage of the disease, it is generally complicated with some partial inversion of the uterus. An inattention to this important fact has led to fatal consequences.

"When a tumour supplied with vessels, and consequently capable of bleeding, grows from an organ so subject to bleed as the uterus, it is difficult to demonstrate whether the hæmorrhage arises from the tumour, or from the uterus. The strong reason for attributing the hæmorrhages to the tumour, is this. As soon as a ligature is applied, and tightened round the stalk, the hæmorrhage from that time ceases, although it may be several days before the tumour comes away.

"It was an opinion of M. Levret, that a polypus did not bleed whilst it remained within the uterus, but that after its expulsion into the vagina, the orifice of the uterus, by constricting its stalk, impeded the return of blood in its veins, which consequently burst and bled profusely. This opinion, however, is contradicted by the foregoing case, as well as others which I shall have occasion to relate: a polypus of the neck or of the orifice of the uterus projects from the beginning into the vagina, consequently it does not undergo that expulsion from the uterus, which takes place in polypus of the fundus, and is capable of being detected from its commencement." 261.

The diagnosis of this disease is a matter of greater difficulty than the treatment. When once detected, any surgeon, with a proper instrument, is competent to remove it. But the case is frequently misunderstood, and our author has known the most experienced practitioners hesitate about the nature of a tumour when detected, and, consequently, about the propriety of removing it.

"As tumours are often found in the vagina, which somewhat resemble polypus, but which are very dissimilar in their nature and treatment, it is important to learn the marks by which they may be distinguished. The tumours which are likely to be mistaken for polypus, are, 1. the prolapsed uterus; 2. the inverted uterus; 3. malignant excrescences from the uterus.

"It is not likely that any man of moderate knowledge and experience should mistake prolapsus for a polypus of the uterus. In prolapsus, the tumour has at its most depending part a palpable orifice, that of the uterus, into which a probe or bougie can be passed several inches; the tumour is sensible, so that if pricked or scratched the patient feels it; the tumour grows broader the higher the finger is passed, and it cannot pass high, for it is soon stopped by the angle where the vagina is attached round to the uterus. The higher the tumour is pushed the easier does the patient become. In all these particulars the polypus is just the opposite; it has no orifice, it is insensible, so that if pricked or scratched the patient does not feel it; the finger can be passed very high, and the higher it is passed the narrower becomes the tumour; the higher the tumour is pushed, the more uneasy becomes the patient. I have seen many cases of this kind which gave occasion to doubts, but never one in which it became a question whether the tumour was prolapsus, or polypus of the uterus.

"Inverted uterus being a rarer occurrence, is less likely to be met with, but when it is, it is more likely to be mistaken for polypus. When the uterus is only partially inverted, that is, when its fundus only is drawn down through its orifice into the vagina, and the patient has survived for many months, the tumour feels exactly like a polypus of the fundus. The distinguishing marks are the time of its first appearance, which must have been immediately after delivery, and its sensibility. In the smoothness of its surface, the roundness of its body, the narrowness of its neck, and its being completely encircled by the orifice of the uterus, it sometimes exactly resembles polypus of the fundus, of which the following case affords an example.

"The first time I saw the patient was in consultation with Dr. Clarke and Dr. Henry Davies; she had been delivered some months before at St. Omer, and immediately after the removal of the placenta, which had been extracted with some violence, a tumour had been felt projecting from the uterus into the vagina, since which she had not only had no hæmorrhages, but had not even ordinary menstruation. When we examined the tumour, we found it about the size of a small apple with a smooth surface, a somewhat narrow stalk, which was completely encircled by the orifice of the uterus, exactly like a polypus, but its quick sensibility to touch, and the circumstances under which it made its first appearance, inclined us to believe that it was an inverted uterus, and not to recommend its removal, particularly as she was losing no blood, and her health was sustaining no injury from it. She returned to the continent, and I did not see her again for two years, when she again came to London, to place herself under the care of Dr. Granville, who had recommended her to submit to an attempt to revert it, and I now saw her in consultation with the doctor. Since my former interview with her, she had become subject to frequent and profuse hæmorrhages, which had bleached her face and broken her health, and it now became an urgent object to afford her relief even at some risk. We agreed, therefore, that the attempt should be made to revert the tumour, but if this failed, which appeared most likely, we proposed to her husband the removal of the tumour by the ligature, stating to him that such an operation had been done successfully, but that it was attended with considerable risk. This both he and the patient were willing to incur; the attempt at reduction failed, but before applying the ligature, her former attendants, Dr. Clarke and Dr. Henry Davies, were consulted, and all of us agreeing to recommend the operation, the ligature was applied by Dr. Clarke; it was tightened every other day, and each time occasioned so much pain as to require a large opiate to quiet it. At length on the fourteenth day both instrument and tumour came away; there were times when I had a strong suspicion that it was a polypus, but a sight of the tumour proved that it was the fundus of the uterus, for it was a hollow cup, the size of a small apple, in the cavity of which could be seen the fallopian tubes. Excepting the pain and some vomiting, the patient had no bad symptoms during the progress of the cure, and several months afterwards her husband called on me to say she was quite well." 266.

A more frequent subject of doubt is, whether the tumour which projects from the uterus into the vagina be a common polypus that admits of removal and a permanent cure, or a malignant excrescence which, if removed, grows again and terminates fatally. On this point, Dr. Gooch speaks more particularly in the second part of the paper on "some unusual Forms of Polypus," to which we shall come in due time. All that he remarks here is this—that "whenever the tumour has a stalk which can be included in a ligature, without danger of including the neck or fundus of the uterus, he would apply it." It succeeds in an immense proportion of cases.

"If polypus of the uterus is overlooked or neglected it ultimately destroys the patient. Frequent hæmorrhages drain the circulation to the lowest point compatible with life, till at length a fresh hæmorrhage occasions a fainting fit or convulsions, in which the patient dies. It is a practical rule, therefore, of vital importance, that whenever hæmorrhages from the uterus resist the ordinary means, the nature of the case should be certified by examination. I have heard of several fatal cases from a neglect of this rule, and many are recorded in books.*

"When hæmorrhages from the uterus arise from a polypus, medicines are useless. The only effectual way to cure the hæmorrhages is to remove the polypus. This may be done either with the knife, as is practised at Paris, or by applying a ligature round the stalk, and tightening it until the tumour falls off. I have never used any other means than the latter, and, as it has served me successfully for many years, and in numerous cases, so that I wish I had as good cure for all diseases, I shall not abandon it for the knife, which, if I may judge from cases which have been related to me, is not always so safe and successful.

"It may be easily supposed, and if an attempt is made, it will soon be found, that to pass a ligature round a tumour, situated in a deep and narrow canal like the vagina, is not an easy task without an instrument adapted for the pur-

* "See Dessault's surgical works, edited by M. Roux, vol. iii. Memoires sur les Polypes. Medico-Chirurg. Journal and Review, for December, 1816. Levret, sur les Polypes de la Matrice, p. 180."

pose.* That which I use consists of two tubes, capable of being separated and joined, and was originally contrived by a German surgeon, of the name of Niessen, but has since undergone many changes in the hands of different surgeons, especially Levret. A representation of its latest form has been copied from Richter's System of Surgery into Mr. Samuel Cooper's First Lines of Surgery. In this sketch the two tubes are curved, to correspond with the curvature of the vagina and sacrum.

"In this instrument I made two changes, the principal one consisted in making the tubes straight instead of curved, the latter form I found unnecessary even with the largest polypi; and it was liable to this great inconvenience, that when the tubes had been passed round the polypus, so as to meet again on the opposite side, if the upper extremities deviated in the slightest degree from each other, (an accident which it was almost impossible to prevent, and which took place notwithstanding their lower extremities were perfectly parallel,) it was impossible to slip up the cross part which was to join them together. On the contrary, if the tubes were straight, it was necessary only to keep the lower extremities perfectly parallel to insure a similar apposition of the upper, and the cross part could be slipped up without any difficulty.

"The instrument which I use for this purpose, and which in numerous cases has assisted me easily through the operation, consists of two silver tubes, each eight inches long, perfectly straight, separate from one another, and open at both ends. A long ligature, consisting of strong whip-cord, is to be passed up the one tube and down the other, so that the middle of the ligature passes across from the upper end of one tube to the upper end of the other, and the two ends of the ligature hang out at the lower ends; the tubes are now to be placed side by side, and, guided by the finger, are to be passed up the vagina, along the polypus, till their upper ends reached that part of the stalk round which the ligature is to be applied; and now the tubes are to be separated, and while one is fixed, the other is to be passed quite round the polypus till it arrives again at its fellow tube, and touches it. It is obvious that a loop of the ligature will thus encircle the stalk. The two tubes are now to be joined, so as to make them form one instrument; for this purpose two rings, joined by their edges, and just large enough to slip over the two tubes, are to be passed up till they reach the upper ends of the tubes which they bind together immoveably. Two similar rings, connected with the upper by a long rod, are slipped over the lower ends of the tubes so as to bind them in like manner; thus these tubes, which at the beginning of the operation were separate, are now fixed together as one instrument. By drawing the ends of the ligatures out at the lower external ends of the tubes, and then twisting and tying them on a part of the instrument which

---

* "How difficult this operation sometimes is if not facilitated by some mechanical contrivance may be seen in the cases published by Dr. Denman. He describes himself, Case the 4th, as making many and strenuous attempts to pass the ligature but without success. At length the ligature was applied, but the patient died before the polypus came away, and appears to have been lost from the unsuccessful attempts of the operation. In Dr. Hunter's museum there is a large polypus, with the statement that after many attempts to pass the ligature the patient died. (See *Denman's Midwifery*, vol. i. p. 100."

projects from the lower rings, the loop round the stalk is thereby tightened, and, like a silk thread round a wart, causes it to die and fall off.

*A* The two silver tubes armed with the ligature, and applied to that part of the stalk which is to be encircled by the ligature; one tube a little separated from the other on its way round the polypus to meet its fellow tube on the opposite side.

*B* The rings which bind the tubes into one instrument, the upper and lower joined together by a long silver rod; on the lower the projection or shoulders.

*C* The two tubes joined together by the upper and lower rings; at the *upper end* a loop of the ligature round the stalk; at the *lower end* the ends of the ligatures twisted round the shoulders of the instrument.

"The instrument being thus adjusted is to be left, but the ligature every night and morning is to be untwisted from the shoulder of the instrument, drawn tighter, and then fixed again round the projecting part, and this is to be done morning and night. As the instrument projects out of the vagina, if the patient was, whilst turning from side to side, to sit down upon it, she might impale herself on it; an accident, which I have heard once took place, and terminated fatally. To prevent this, the late Dr. Clarke contrived a round flat wooden shield, which is fixed to his instrument so close to the outer orifice that even if the patient was to sit down on the instrument it could not be thrust higher in the vagina. I have always satisfied myself with making the patient understand the necessity for care in turning, but such a guard could easily be adapted to my instrument; if the projecting part or shoulders were made two inches broader, they would answer the purpose." 273.

The only danger attendant on the operation is, that the ligature may include a portion of the uterus. In one of Dr. Denman's operations of this kind, pain and sickness quickly followed the application of the ligature—and as soon as the latter was slackened, these symptoms ceased. They were again renewed whenever he attempted to tighten the ligature. The patient died about six weeks afterwards, and, on opening the body, it was discovered that the uterus was inverted, and that the ligature had included the inverted portion. Mr. Abernethy relates in his lectures several cases, where women had died from the ligature of polypus uteri. The same accident happened in the practice of Dr. William Hunter, and he relates it as a warning to others.

III. *Case.* A poor woman, about 40 years of age, was received into Bartholomew's Hospital, having been delivered by means of the forceps six months previously. She had now a tumour in the vagina, which protruded in the perpendicular posture, but was easily returned. It was as large as the head of a new-born infant, and was attached by a stalk, nearly as thick as the wrist, to the usual seat of the cervix uteri. The orifice of the uterus could not be felt. The tumour was of a pale flesh-colour, had a knotty surface, and felt firm. The patient had a profuse colourless discharge, but she had no hæmorrhages, and had for some time ceased to menstruate. The ligature was applied round what was supposed to be the stalk of the tumour. It occasioned little pain when first applied, but, towards evening it became so severe as to resemble labour. This was relieved by an opiate and she passed a comfortable night. Next

The most convenient mode of preparing the instrument for the operation. *A* the two tubes armed with the ligature ready to pass up to the stalk of the polypus, and still separable so as to pass round it. *B* the upper and lower rings, connected by a silver rod, already slipped up over the long outer ends of the ligature, and ready to be slipped up over the two tubes as soon as they have been passed round the polypus, so as to encircle the stalk with the ligature; the ends of the ligature made into a knot, to prevent this part of the instrument dropping off during the operation.

day the pain increased, extending up the loins and down the limbs. The ligature was tightened every day, with a recurrence of the pain, requiring an opiate. The tumour became livid and the discharge fetid. On the seventh day a violent hæmorrhage came on, accompanied by death-like faintings and cold sweats. The hæmorrhage was arrested by local astringents, and the fainting relieved by brandy and ammonia. But the symptoms, though mitigated, continued, and she died on the fifteenth day after the operation. On opening the body, the uterus was found of its natural size and structure—the tumour grew from the orifice of the uterus all round, so as to be continuous with the cervix, and so as to cover the aperture of the uterus, and make it impossible to say where the neck of the uterus ended and stalk of the tumour began. The ligature had been applied so high as to include the projecting neck of the uterus—the posterior part of it had occasioned ulceration into the cavity of the peritoneum, in which there was an aperture of about an inch in extent. The inner structure of the tumour was similar to that of fleshy tubercle—there was no inflammation of the peritoneum.

The danger of including the uterus in the ligature may be avoided, Dr. Gooch thinks, by attention to the following rules.

"1st. Instead of aiming at passing the ligature as high as possible on the stalk, to pass it as low as possible, taking care to pass it over the body of the tumour. It is true by these means a portion of the stalk will be left above the ligature; but I know by experience that it does not grow again; like the remnant of the umbilical chord, it dies and falls away. These tumours have little life, and die above as well as below the ligature. By a case, which I shall soon relate, it will be shown, that this is not a matter of probability, but of certainty. 2nd. When the stalk grows from the cervix, if the os uteri can be felt, it will be the best guide where the neck ends and the stalk begins; the ligature ought to be applied a little below the orifice, but if it cannot be felt, the next best guide is the ordinary length of the projecting part of the neck, that is, about two-thirds of an inch. When the polypus is very large, and the vagina closely contracted, it is difficult or impossible to reach the stalk and the cervix, so as to make any thing like an accurate measurement, and the first rule only is practicable. 3d. To attend to the sensations of the patient when the ligature is tightened; if it gives much pain, there is every reason to believe that it has included a part of the uterus." 278.

It generally requires from two to ten days for the ligature to make its way through, being tightened night and morning. The discharge becomes more fetid from day to day, and demands great attention to cleanliness. The operation is generally successful—and a disease which has resisted remedies for years is removed in a week. The hæmorrhages which had lasted so long and occasioned so much debility, suddenly cease—and the patient rapidly recovers her health. Sometimes, however, this requires to be assisted by tonics and the usual restorative medicines. It has been supposed by some writers that a ligature could not be applied to a thick-stalked uterus; but this, our author avers, is a mistake. He has repeatedly applied the ligature to very thick-necked polypi, with no other inconvenience than that the ligature was many days in making its way through.

Several cases in illustration are related by Dr. Gooch, and then he makes some observations on certain excrescences—more especially the cauliflower excrescence—which grow from the uterus, and are liable to be mistaken for polypus. The author comes to the following practical conclusion.

"That these fungous excrescences described by Levret, Herbiniaux, and Dr. Clarke, which I have found in the uterus and in the vagina, and which agree in these leading properties, that instead of a dense firm substance they are of a spongy or vascular structure; that if removed they grow again and kill by producing frequent and profuse hæmorrhages; are only the same growth in different parts of the genital cavity, and are specimens of the same disease which, in other parts of the body, is best known in this country under the denomination of the bleeding fungus or fungus hæmatodes.

"In all these cases of fungus excrescence in the vagina, the best practical rule I believe to be this; whenever the form of the excrescence is such that the whole can be removed by a ligature, without including any portion of the uterus, apply it, distinctly stating to the patient or her friends that it is not done with the same confidence of success as in a common polypus, but as the only remedy which gives the patient any chance of life, and if it fails by the excrescence growing again, it does not render the case worse than it was before.

"I do not believe that any man can tell infallibly by touch whether a tumour in the vagina is a malignant excrescence, which is to grow again, or a benign one, which, if removed, will never return. A rough uneven surface is no test. The polypus described in Case No. III. which turned out to be a common polypus in structure, and which would have been successfully removed if the ligature had been applied an inch lower, had a rough surface; and I have successfully and permanently removed tumours which, because they had uneven surfaces, had been judged by other practitioners to be malignant excrescences. It is a prevalent notion among medical men, that these malignant excrescences are far more common than they really are. Among the cases about which I am consulted, especially from the country, in which disease of structure is apprehended in the uterus, no one is so often named as the cauliflower excrescence. If the surface of the tumour or even the neck of the uterus is a little irregular, if blood follows an examination, and the patient states that she has a watery discharge, by which she means little more than that it is colourless, all which are common occurrences in the diseases of structure in this organ, the case is sure to be set down as cauliflower excrescence. If these suspicions were accurate this disease would be the most common of the diseases of this organ, yet, the fact is, that it is the most rare. Where we see one case of cauliflower excrescence we see ten or even twenty of common polypus and fifty of carcinoma or malignant ulcer of the uterus." 309.

We have now given a very full analysis of every article in Dr. Gooch's volume, with the exception of a long one at the end, re-published from the Quarterly Review—and which is itself a review of the long-litigated subject of the plague. It is a very clever article; but we think Dr. Gooch might have allowed it to remain anonymous where it was first published. It was rather foreign to our author's usual professional pursuits; but it shews that a man of talent and erudition may write well on a subject of which he has no personal or practical knowledge. We need not add to the approbation which we have so often expressed of Dr. Gooch's work. We have reason to know that a large edition is exhausted already, and we can have no doubt that it will become a standard publication in the library of every respectable practitioner, whether physician or surgeon, or both.

## Figures and Tables

**Figure f1:**
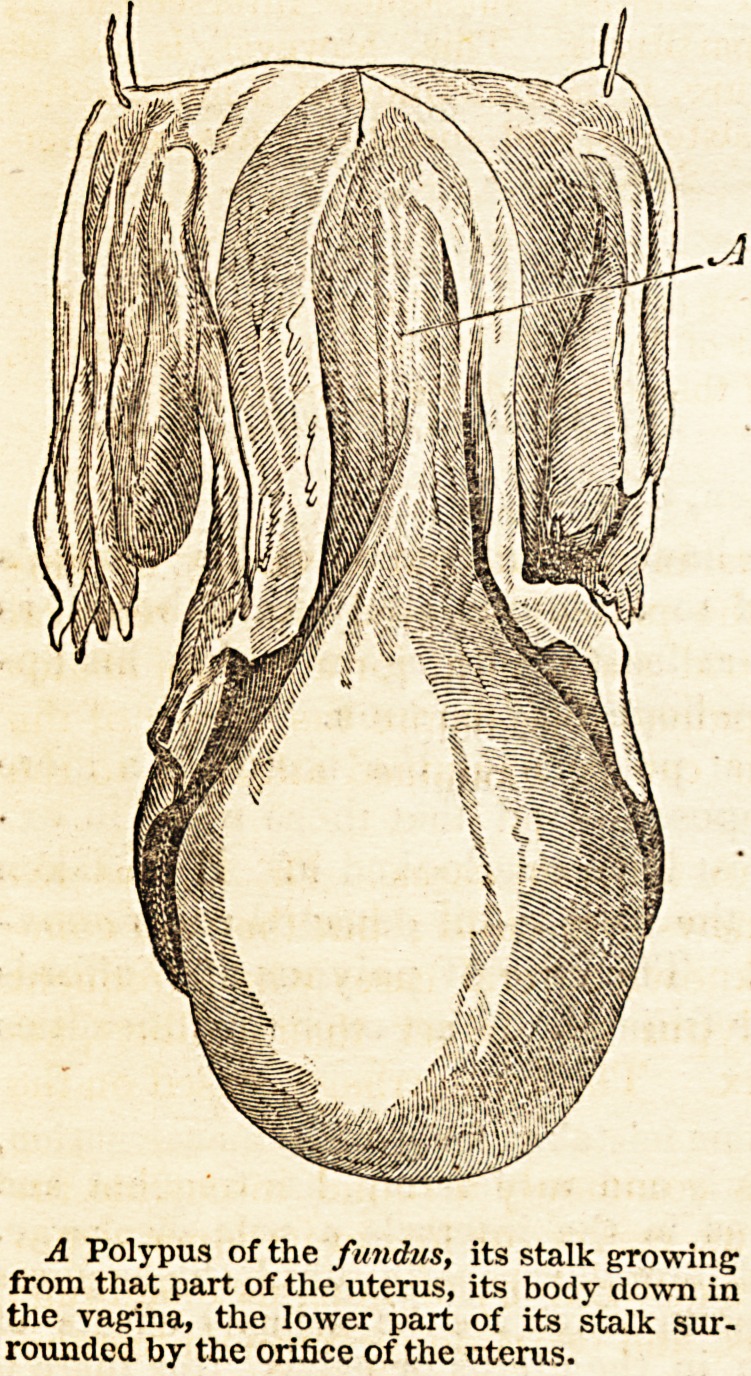


**Figure f2:**
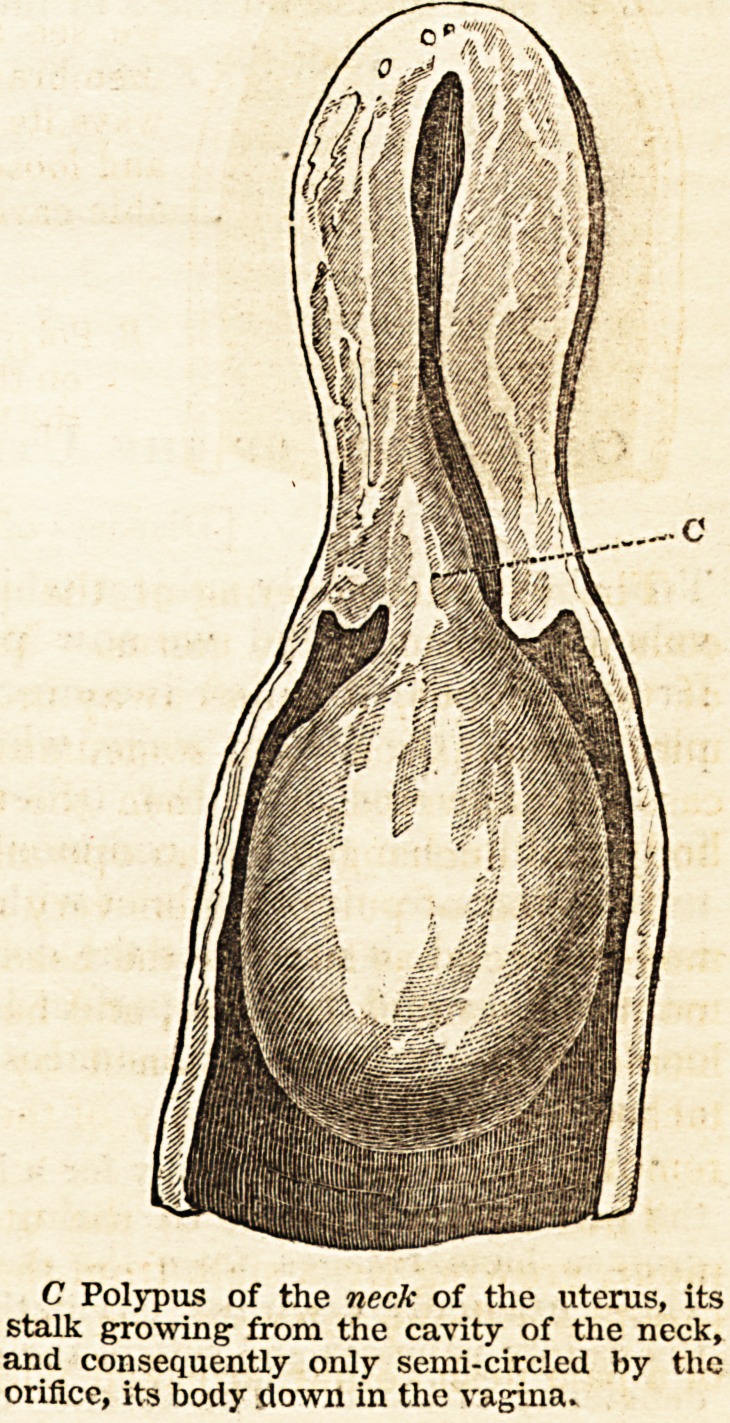


**Figure f3:**
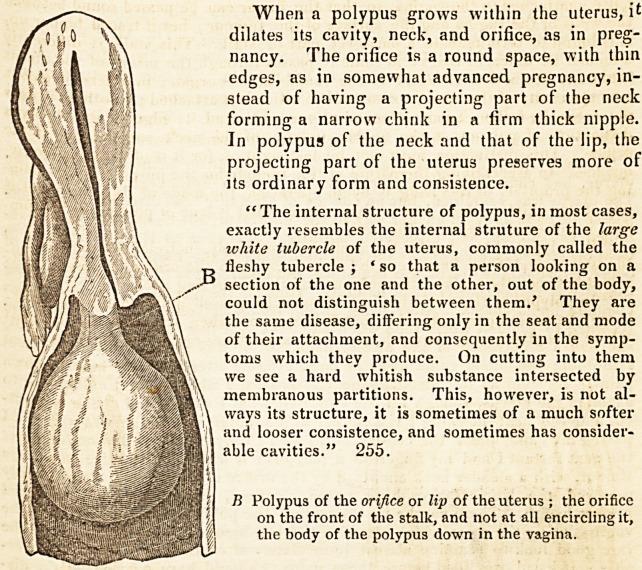


**Figure f4:**
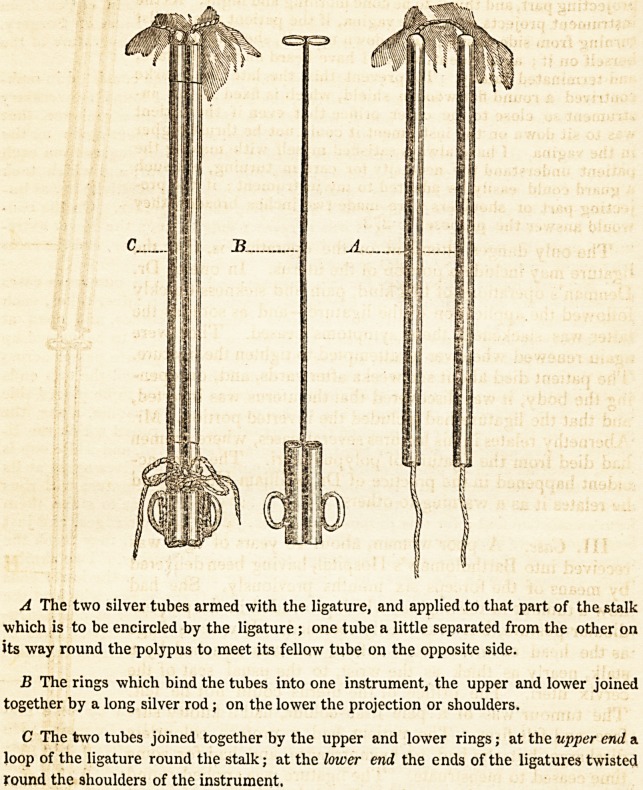


**Figure f5:**